# Mucopolysaccharidoses I and II: Brief Review of Therapeutic Options and Supportive/Palliative Therapies

**DOI:** 10.1155/2020/2408402

**Published:** 2020-12-04

**Authors:** Haiyan Nan, Chanbum Park, Sungho Maeng

**Affiliations:** Graduate School of East-West Medical Science, Kyung Hee University, Yongin, Gyeonggi-do 17104, Republic of Korea

## Abstract

*Purpose*. Mucopolysaccharidoses (MPS) are group of inherited lysosomal storage diseases caused by mutations of enzymes involved in catalyzing different glycosaminoglycans (GAGs). MPS I and MPS II exhibit both somatic and neurological symptoms with a relatively high disease incidence. Hematopoietic stem cell therapy (HSCT) and intravenous enzyme replacement therapy (ERT) have had a significant impact on the treatment and comprehension of disease. This review is aimed at providing a comprehensive evaluation of the pros and cons of HSCT and ERT, as well as an up-to-date knowledge of new drugs under development. In addition, multiple disease management strategies for the uncontrollable manifestations of MPS I and MPS II to improve patients' quality of life are presented. *Findings*. Natural history of MPS I and MPS II shows that somatic and neurological symptoms occur earlier in severe forms of MPS I than in MPS II. ERT increases life expectancy and alleviates some of the somatic symptoms, but musculoskeletal, ophthalmological, and central nervous system (CNS) manifestations are not controlled. Additionally, life-long treatment burdens and immunogenicity restriction are unintended consequences of ERT application. HSCT, another treatment method, is effective in controlling the CNS symptoms and hence has been adopted as the standard treatment for severe types of MPS I. However, it is ineffective in MPS II, which can be explained by the relatively late diagnosis. In addition, several factors such as transplant age limits or graft-versus-host disease in HSCT have limited its application for patients. Novel therapies, including BBB-penetrable-ERT, gene therapy, and substrate reduction therapy, are under development to control currently unmanageable manifestations. BBB-penetrable-ERT is being studied comprehensively in the hopes of being used in the near future as a method to effectively control CNS symptoms. Gene therapy has the potential to “cure” the disease with a one-time treatment rather than just alleviate symptoms, which makes it an attractive treatment strategy. Several clinical studies on gene therapy reveal that delivering genes directly into the brain achieves better results than intravenous administration in patients with neurological symptoms. Considering new drugs are still in clinical stage, disease management with close monitoring and supportive/palliative therapy is of great importance for the time being. Proper rehabilitation therapy, including physical and occupational therapy, surgical intervention, or medications, can benefit patients with uncontrolled musculoskeletal, respiratory, ophthalmological, and neurological manifestations.

## 1. Introduction

Mucopolysaccharidoses (MPS) are rare, heterogeneous group of lysosomal storage disorders caused by a deficiency of various catalyzing enzymes that break down polysaccharides, called glycosaminoglycans (GAGs). GAGs are ubiquitously present in the connective tissue and play important roles in cell growth and proliferation, cell surface binding, and histamine storage [1–6]. GAGs are classified by their different core disaccharide structures into dermatan sulfate (DS), heparan sulfate (HS), keratan sulfate (KS), and chondroitin sulfate (CS). DS is the main constituent of conjunctive tissues; HS is a major component of cellular membranes; KS and CS are major component of the cartilage and cornea [7].

After Charles Hunter and Gertrud Hurler first reported MPS in patients in 1917 and 1919, with the metabolic disorders now bearing their names (MPS I: Hurler syndrome, MPS II: Hunter syndrome), subsequent MPS types have been assigned numbers and eponyms loosely associated with the chronology and origin of their report [8]. Eleven enzymatic deficits are known to be responsible for seven different types of MPS along with the identification of the gene mutations responsible for the disease in the 1970s [9]. Each MPS disorder is caused by a deficiency in the activity of a single, specific lysosomal enzyme required for GAG degradation [10]. See Figure S1 and Table S1 in the Supplementary Materials for the stepwise degradation of the main GAG chains and defective enzymes and accumulated GAG for each type of MPS.

The majority of MPS is inherited in an autosomal recessive manner, except for MPS II, which is an X-linked recessive disease [11, 12]. The incidence of overall MPS is approximately 1 : 25,000 births, and it varies between different MPS types. The estimated incidence of MPS I is 0.69-1.66 per 100,000 live births, while the incidence of MPS II is 0.30-0.71 per 100,000 live births worldwide [13–21]. The incidence rate has not been calculated for MPS IX because only four cases have been reported so far worldwide. MPS incidence also shows regional differences. The incidence of MPS I and MPS III is higher in European countries, whereas MPS II is the most prevalent type in Asian countries, including Japan, South Korea, China, and Taiwan [16, 22–26]. In South Korea and Japan, MPS II accounts for more than 50% of all MPS types, followed by MPS I and MPS III [24, 27]. In Denmark and Norway, MPS I is the most common type, which accounts for 30% and 60% of MPS patents, respectively [10, 22].

GAG accumulation in various tissues and organs leads to a wide spectrum of clinical manifestations and high progression rates within MPS types [13]. Somatic manifestations of MPS may include coarse facial features, hepatosplenomegaly, obstructive and restrictive respiratory disease, cardiac valve disease, musculoskeletal abnormalities, impaired vision, and dental abnormalities. These somatic symptoms are found in MPS types I, II, IV, VI, and VII [13, 28, 29]. Neurological symptoms with clinical features of aggressive, hyper/overactive behavior, developmental delay, and cognition decline are observed in patients with MPS I, II, III, and VII, the types associated with HS accumulation [30–32].

These lysosomal enzyme deficiencies are biochemically characterized by increased GAG concentration in urine, blood, and cerebral spinal fluid [33]. The diagnosis of MPS starts with the assessment of GAG in the urine (qualitative and quantitative) in suspected patients. A positive result is very suggestive of an MPS, but false-negative results are common. A confirmative diagnosis requires an enzyme activity assay in leukocytes, fibroblasts, dried blood spots, or plasma using substrates specific for the enzyme deficient in each MPS type, followed by further clinical, molecular, and biochemical analyses [9]. Given the clinical heterogeneity and progressive worsening characteristics of the MPS, it is important to establish a diagnosis as early as possible to initiate intervention before irreversible damage occurs. Due to the crucial role of early detection, the necessity of newborn screening has been raised. A direct multiplex assay of lysosomal enzymes in dried blood spots on filter paper by use of tandem mass spectrometry and a multiplexed immune-quantification assay of lysosomal proteins from dried blood spots on filter paper have been developed in 2006 [34, 35]. In addition, a high-throughput multiplex method with high-performance liquid chromatography-tandem mass spectrometry to simultaneously assay several lysosomal enzymes for MPS in blood samples has been developed in the year 2013 [36]. IDUA enzyme activity screening in newborns is currently being performed as an initial pilot program in the United States, Taiwan, Italy, Austria, and Hungary [37–41].

MPS I and MPS II are the MPS types that display both somatic and neurological symptoms (including neurocognitive retardation and development delay) and exhibit relatively high incidence rate. For these reasons, MPS I and MPS II are chosen to provide the most recent knowledge on disease management. This paper reviews the appearance and progression of neurological, musculoskeletal, and ocular signs and symptoms in patients with MPS I and MPS II with a specific focus on disease treatment and management. It evaluates emerging therapies targeting uncontrolled manifestations of MPS, including blood-brain barrier- (BBB-) penetrable ERT, substrate reduction therapy (SRT), and gene therapy. Furthermore, it demonstrates the application of supportive/palliative therapies, such as physical and occupational therapy, hydrotherapy, surgical intervention, and medications which control the unmanageable symptoms associated with the musculoskeletal, respiratory, optical, and neurological symptoms of MPS.

## 2. Results and Discussion

### 2.1. Natural History of MPS I and MPS II

MPS I is an autosomal recessive disorder caused by the deficiency of *α*-L-iduronidase (IDUA), the lysosomal enzyme required for the degradation of DS and HS. This enzyme deficiency leads to HS and DS accumulation in the lysosomes of cells and extracellular tissues and organs. At least 257 variations have been identified with poor genotype/phenotype correlation [9]. Homozygosity or compound heterozygosity for two common nonsense mutations, p.W402X and p.Q70X, predominates among severe phenotypes, whereas p.P533R is associated with an intermediate-to-severe phenotype [37, 42]. Typically, MPS I has been classified into three phenotypes: Hurler syndrome, Hurler-Scheie syndrome, and Scheie syndrome. Hurler syndrome is the most severe form of the three phenotypes, whereas Scheie syndrome is the mildest [43, 44]. The varying degrees of residual enzyme activity and GAG accumulation throughout the body result in a wide spectrum of disease manifestations and severity.

Table 1 summarizes the prevalence and onset time of clinical symptoms in Hurler syndrome [30, 45–51]. Coarse facial features, corneal clouding, hepatosplenomegaly, kyphosis/scoliosis, and cardiac valve disease are the most common symptoms in Hurler syndrome, occurring in 86.4%, 70.9%, 70.0%, 70.0%, and 48.9% of the patients, respectively. The median age at each symptom onset is 0.9 years, 1.1 years, 1.1 years, 1.0 years, and 1.3 years, respectively [45, 46]. Hurler syndrome is associated with significant developmental delay and cognitive impairment. Because the symptoms occur shortly after birth and progress rapidly, most Hurler patients die within the first decade of life. Conversely, Scheie syndrome has typically milder symptoms, slower disease progression, and late onset symptoms, typically between the ages of 3 and 7 years. Although patients with Scheie syndrome usually develop significant disease-related morbidities, they still have normal cognitive function and will likely survive into adulthood. Hurler-Scheie syndrome is an intermediate phenotype characterized by mild-to-no cognitive impairment but it exhibits somatic symptoms that reduce life expectancy into the second or third decade of life [46].

MPS II is an X-linked lysosomal storage disorder caused by deleterious mutations in the iduronate-2-sulfatase (I2S) gene. Enzyme deficiency results in the lack of degradation of DS and HS and therefore their progressive accumulation throughout the body. To date, 628 variations of I2S have been identified. Among them are missense/nonsense variants, which represent about 50% of cases, and small deletions, which represent about 29% of cases [9, 24]. A few recurrent mutations, such as G374G, R443X, L522P, and recombination mutations, are found in MPS II patients [24, 52].

The clinical presentations of MPS II lie in wide spectrum of symptoms, from the most severe symptom to the mildest symptoms. Approximately two-thirds of patients with severe cases of MPS II exhibit neuropathic symptoms. Table 1 summarizes the prevalence and onset time of clinical symptoms in the severe form of Hunter syndrome [30, 45, 47, 48, 53–58]. This severe type shows developmental delay and cognitive impairment, which becomes apparent at 3.2 years of age, followed by a rapid decline that becomes apparent from 4 years of age. The severe form of the disease progresses rapidly, and most patients die in their twenties [42, 59]. Coarse facial features, hepatosplenomegaly, airway diseases, joint contractures, behavior problems, and cognition impairment are the most common symptoms in Hunter syndrome, occurring in 95%, 89%, 70%, 84%, 73%, and 100% of patients, respectively. The median ages of symptom onset are 2.4 years, 2.8 years, 3.4 years, 3.6 years, 4 years, and 3.2 years, respectively [45, 46]. The mild type of Hunter syndrome is more likely to have somatic symptoms without cognitive impairment. However, other neurological symptoms including seizures or myelopathy may occur in these patients. The mild type has delayed symptom onset, and patients usually live into adulthood [42, 59].

Natural history of severe types of MPS I and II demonstrates that pathological progression occurs much faster in MPS I than MPS II because both somatic and central nervous system (CNS) symptoms occur relatively later in MPS II. Additionally, MPS I and MPS II symptoms exhibit different patterns. While ocular manifestations (corneal clouding and retinopathy) are uncommon in MPS II, they occur in about 80% of MPS I patients. Joint contraction is more frequent in MPS II (84% prevalence) than in MPS I (38% prevalence). Kyphosis/scoliosis is more prevalent in MPS I than in MPS II, occurring in 70% and 39% of patients, respectively.

Despite the fact that early and accurate diagnosis in MPS patients is highly important, data from the MPS I registry reveals a delay between symptom onset and disease diagnosis. In Hurler syndrome, initial symptoms occur at a median age of 6 months, but patients only receive an initial diagnosis at a median age of 12 months. Patients with Hurler-Scheie and Scheie syndromes show first symptoms sometimes after infancy, and there is a 2- to 4-year gap between symptom onset and diagnosis [46]. Data from the MPS II registry shows that the median age of symptom onset is at 1.5 years, but the syndrome diagnosis is at 3.5 years. This shows an approximate two-year delay between symptom onset to diagnosis in Hunter syndrome [53]. The lag in symptom onset and diagnosis time may be due to the rarity of the disease, variability in clinical presentation, different disease progression states, and the nonspecific nature of some of the initial manifestations of the disease.

### 2.2. Current Therapies: Efficacy and Limitations

Over the last few decades, there has been considerable development in the availability of disease-specific treatments. Nowadays, therapies for MPS I and MPS II involve hematopoietic stem cell therapy (HSCT) and intravenous enzyme replacement therapy (ERT), which have had a significant impact on the treatment and comprehension of disease. After the introduction of HSCT and ERT, the natural history of MPS I and MPS II has changed significantly [60].

#### 2.2.1. Hematopoietic Stem Cell Therapy

Hematopoietic stem cells are created in the bone marrow and are also found in peripheral blood and umbilical cord blood. After undergoing immunosuppressive therapy to deplete patient's immune response, the patient receives healthy matched donor cell transplant, and the enzymes are secreted from the donor cell [61]. HSCT may provide a permanent source of the missing enzyme by engrafted donor-derived hematopoietic stem cells. Additionally, HSCT enables the engraftment of donor-derived microglial cells to produce the deficient enzyme in the brain locally. This treatment, which is different from the intravenous administration of enzymes, may be effective in treating CNS manifestations [62]. HSCT needs to be performed only once, and the effect has long-lasting benefits for both cognitive function and physical symptoms [63, 64]. After the first successful bone marrow transplant in 1980, approximately 600 patients with a severe phenotype of MPS I have received HSCT [61, 65]. HSCT has been reported to prevent many of the clinical symptoms of MPS I, VI, and VII [66–69]. It can alleviate symptoms of growth, endurance, hepatosplenomegaly, joint mobility, upper airway obstruction, and respiratory function, but it shows a limited effect on existing skeletal dysplasia and cardiovascular abnormalities [43, 70]. One recent study showed that the overall survival rate of patients with Hurler syndrome undergoing HSCT at 1 year and 20 years was the same at 73.7%. Meanwhile, another study reported that the survival rates at 1 year and 25 years were 70% and 37%, respectively [71, 72]. This implies a reduced mortality rate and increased life expectancy in MPS I patients received HSCT.

HSCT possesses the advantage of only having to be performed once with life-long effect of continuous enzyme provision from donor stem cells. However, the success of HSCT depends on many factors including the transplantation age, cardiopulmonary status, type of donor, and the ability to achieve stable engraftment without the development of graft-versus-host disease [73]. Hematopoiesis provides a variable enzyme level, thus differing HSCT efficacy in individual patients [74, 75]. Furthermore, transplantation procedures performed too late may be unsuccessful in treating preexisting damage [76]. Although HSCT may improve clinical manifestations of the disease, it does not seem to correct these manifestations entirely and does not affect preexisting cognitive impairment. Meanwhile, HSCT is not able to cure symptoms in the bone or cornea due to insufficient delivery of enzymes to the sites [43, 70]. Therefore, HSCT is reserved for patients below the age of 2.5 with the most severe type of MPS and constitutes the standard care for treating severely affected MPS I patient with a developmental quotient >70% of the normal, with an increased likelihood of maintaining cognitive abilities with early intervention [66]. Pretransplant ERT is recommended after diagnosis and before HSCT to optimize organ function and reduce morbidity and mortality [77].

It is noted that HSCT is not recommended for MPS II, which is the type that possesses similar clinical presentation of both somatic and neuropathic symptoms as MPS I. This is due to the fact that previous data has found no evidence of neurocognitive stabilization [78]. The difference effect of HSCT in MPS I and MPS II may be explained by the different times of diagnosis, with earlier diagnosis of MPS I as early as 12 months and relatively late diagnosis of MPS II at 3.5 years. Because HSCT can preserve intellectual development for most children—who would otherwise develop severe cognitive impairment—if applied early [67], it may also benefit MPS II patients receiving early diagnosis with newborn screening in the future.

#### 2.2.2. Enzyme Replacement Therapy

ERT is the regular administration of genetically engineered enzymes obtained through recombinant DNA technology, which is a method to compensate for the defective enzyme. Infused enzymes are taken up by the cells into the lysosomes to catalyze accumulated GAGs, which finally lead to symptom resolution. ERT regimen involves the intravenous infusion of the recombinant human enzyme weekly or every other week. Currently, ERT has been developed for patients with MPS I and MPS II: *laronidase* (Aldurazyme®, BioMarin) was approved in 2003 for MPS I [79, 80], and *idursulfase* (Elaprase®; Shire Human Genetic Therapies, recently acquired by Takeda Pharmaceutical) and *idursulfase-beta* (Hunterase®, GC Pharma) were approved in 2006 and 2012 for MPS II [24, 81, 82]. ERT has benefited patients in terms of improving joint mobility, walking ability, and pulmonary and respiratory functions, while also reducing spleen and liver volume in confirmatory clinical trials [83]. Some studies have reported that ERT increased the patient survival rate [84].

Generally, ERTs for MPSs have an acceptable safety profile and have benefited patients in alleviating several symptoms. However, the following factors have limited ERT application for MPS patients. First, the human body recognizes ERT as “foreign” and thus produces antibodies upon drug administration that may neutralize the ERT effect and induce life-threatening anaphylactic drug allergy. Up to 90% of the patients with MPS I and 50% of the patients with MPS II experience an initial infusion reaction, which can be resolved after months with antipyretics and/or antihistamines [85].

Second, ERT is burdensome for the patients and their families due to the life-long requirement of weekly or biweekly intravenous administration. Furthermore, although annual cost for ERT differs slightly depending on the country, it is quite expensive, which makes it unaffordable to many patients. One report indicated that the annual cost of ERT for an MPS I patient is £258,201 for an adult and £139,563 for a child, whereas it is £537,605 for an adult and £314,004 for a child with MPS II [86]. Although many countries, such as the United States, UK, Japan, South Korea, Brazil, Russia, and Malaysia, cover ERT cost via national reimbursement or national tender system, there are still many countries where patients have to pay out of pocket for the treatments, therefore, restricting the accessibility of ERT.

Last but not least, although many patients present neuropathic symptoms and are classified as severe type of MPS, ERT does not work for these patients, and it is thus regarded as “partial cure” treatment. Meanwhile, ERT shows little effect on joint/skeletal symptoms, heart valve disease, and corneal opacity [12, 48, 60, 62, 83, 87, 88]. These observations can be explained by two reasons. First, the high-molecular weight enzymes cannot easily penetrate the bone, cartilage, or BBB via intravenous (IV) administration. Moreover, injected enzymes are less effective in ocular pathologies due to the retina-brain barrier and the avascular nature of the cornea [84]. Secondly, the expression level of the mannose-6-phosphate (M6P) receptor, which transports enzymes into the cells and then into the lysosomes, varies in different tissues. While the expression level is high in the heart, lung, and kidney, they are low in the muscle and brain [89, 90]. These observations may explain penetration problems at specific sites and limited effects on joint/skeletal symptoms, heart valve disease, optic symptoms, and neurologic symptoms. In these circumstances, the necessity of new therapeutic strategies has been raised.

### 2.3. Novel Therapies under Development

As previously mentioned, unmanaged manifestations of MPS I and MPS II include neuropathic CNS symptoms, musculoskeletal dysfunction, and ophthalmological disorders. Novel therapies that are aimed at curing the disease with the addition of supportive/palliative treatment are thoroughly evaluated in the following sections.

Several drugs that will hopefully address manifestations that do not respond well to ERT or HSCT are in the preclinical or clinical phases of development.  Table 2 summarizes the new and promising drugs under development for MPS I and MPS II [62, 87, 91, 92]. Most of the new therapies target the neuropathic symptoms by delivering enzymes or genes into the brain of patients with MPS I and MPS II. These approaches include (1) BBB-penetrable ERT, which modifies the ERT for BBB penetrability, (2) gene therapy, which corrects the defective gene in the genome by delivering functional genes via virus vector or genome editing technology, and (3) SRT, which reduces GAGs accumulation by decreasing their synthesis or increasing lysosome function.

#### 2.3.1. BBB-Penetrable ERT

The BBB's control of vascular permeability is essential in preventing neurotoxic substances and microorganisms from invading the CNS. However, it impedes the delivery of high-molecular weight therapeutic agents into the brain. Due to the difficulties in BBB penetration of the IV-administered ERT, changing the administration routes or redesigning the enzymes that could penetrate into the BBB could be considered.

Clinical studies are exploring alternative administration routes, such as the intrathecal (IT) or intracerebroventricular (ICV) routes. A phase I clinical trial of *iduronidase*-IT administered in tandem with standard dual HSCT therapy with peritransplant intravenous ERT reported positive results in 2019 (ClinicalTrials.gov Identifier (NCT): NCT00638547) [25]. A group of patients with Hurler syndrome who received *iduronidase-*IT in addition to HSCT and ERT showed significant decreases in cerebrospinal fluid (CSF) opening pressure, markers of disease activity, and markers of inflammation [90]. A phase I/II clinical trial performed in a group of randomized MPS II patients administrated 1 mg, 10 mg, and 30 mg of *idursulfase*-IT monthly for 6 months along with *idursulfase*-IV weekly, reported 80~90% GAG decrease in CSF in 2015 [89]. This promising result led to a phase II/III initiation with monthly administration of 10 mg *idursulfase*-IT in tandem with *idursulfase*-IV weekly in order to determine the effect on neurodevelopmental status in patients with severe types of MPS II (NCT02055118). However, per Shire (SME-Medical Communications, August 2017), there was no difference in cognition between the *idursulfase*-IT treated group and the control group after 12 months of treatment. Currently, they are performing a 10-year extension study of the phase II/III to further evaluate *idursulfase*-IT for long-term safety and cognition impairment (NCT02412787). The efficacy of *idursulfase-beta* via ICV route to reduce HS content in the CSF was reported in the MPS II murine model in 2018 [93], and a phase I/II clinical study administering *idursulfase-beta*-ICV in combination with approved ERT-IV administration in 6 paediatric patients was completed in 2019 [87]. In April 2020, GC Pharma and Clinigen announced the submission of New Drug Application in Japan for *idursulfase*-beta-ICV after receiving the Orphan Drug Designation grant in March (Press Release, Clinigen, April 01, 2020). It is expected that these alternative routes of ERT administration will benefit patients with currently unmanaged CNS symptoms.

Another strategy to control the neurological symptoms is the modification of the enzymes for BBB penetrability. BBB penetration requires the enzymes to cross a physiological transport system localized within the BBB. This system is responsible for the transport of molecules from the blood to the brain. The BBB-penetrable ERT approach involves receptor-mediated transcytosis, a process by which several endogenous proteins (e.g., insulin, leptin, and transferrin) bind to their specific receptors on capillary endothelial cells of the brain to transport into the brain parenchyma. Enzymes fused with antibodies for these receptors are sufficient for endocytosis into the endothelial cells, and they are followed by exocytosis into the abluminal space in the brain. Accordingly, deficient enzymes in each MPS type can be delivered into the brain parenchyma via the blood within the vascular lumen to exert their efficacy towards the target neuron cells [87].

Each company has adopted different strategies to enhance BBB penetration. JCR Pharmaceuticals combined anti-human transferrin receptor antibodies with *α*-L-iduronidase (Project Code: JR-171) or iduronate-2-sulfatase (Project Code: JR-141) to enable the BBB penetration via IV administration [87]. A phase I/II study of JR-171 weekly administration in MPS I patients is in progress (NCT04227600). A phase I/II study of weekly administration of JR-141 in patients with MPS II (NCT03128593) reported HS and DS suppression in plasma and urine during a 4-week treatment period with a significant decrease of HS in the CSF at the 3-week time point [92]. Currently, JCR is performing an open-label phase II/III study in MPS II patients in order to evaluate the safety and efficacy of JR-141 during a 1-year treatment (NCT03568175). Another pharmaceutical company, ArmaGen Technologies, conjugated the anti-human insulin receptor antibody with *α*-L-iduronidase (Project Code: AGT-181) and iduronate-2-sulfatase (Project Code: AGT-182) to enable sufficient enzyme delivery to the brain [87]. The results from a phase I/II clinical study of AGT-181 IV administration in MPS I children showed stabilization of the neurocognitive development quotient and cortical grey matter measurement after 52 weeks of treatment (NCT03053089, NCT03071341), [94]. A phase I safety and dose-finding study of AGT-182 weekly IV administration (NCT02262338) was completed in 2017, but the data yet to be published. Meanwhile, Denali Therapeutics combined the antibody binding site of anti-human transferrin receptors with IDS to increase brain uptake of enzyme (Project Code: DNL-310) and is performing a phase I/II trial in MPS II children with weekly DNL-310 IV administration over a 6-month period (NCT04251026).

Apart from the receptor-mediated transcytosis strategy to penetrate the BBB, another approach involves combining a chemical transporter with proteins to facilitate the transport of enzymes into the cells and lysosomes by binding to the cell-surface heparan sulfate receptors. TEGA Therapeutics conjugated recombinant enzyme with a carrier—a guanidinylated form of neomycin (GNeo). This carrier possesses a high affinity for cell-surface heparan sulfate proteoglycans and has the capacity to take up the macromolecular cargo through micropinocytosis, which enables enzyme delivery to lysosomes. As reported in MPS murine model in 2017, the intranasal delivery of GNeo-conjugated *α*-L-iduronidase resulted in the reduction of GAG storage in the brain [91].

BBB-penetrable ERT is a promising strategy to control CNS symptoms, including cognitive impairment, developmental delay, behavior problems, and seizures, which are unmanaged by current ERT.

#### 2.3.2. Gene Therapy

The concept of gene therapy is to provide a functional copy of the defective gene, which will permanently reside within the cells and slow or reverse the disease state. Considering each MPS type is a result of a single-gene mutation of the lysosomal enzyme, delivering the therapeutic gene into the cells of multiple organs will enable dramatic improvement throughout the body. A variety of gene therapies with different viral vectors, such as adenovirus (AV), adeno-associated virus (AAV), retrovirus (RV), and lentivirus (LV), are under investigation in preclinical or clinical stages with different administration routes: systemic IV administration or direct administration into the CNS via intracerebral injection and intra-CSF injection [62]. AAV vectors have some advantages in terms of long-term gene expression and safety profile compared with other viral vectors. As AAV vector genomes exist as episomes in target cells and rarely integrate into the genome, there is a lower risk of integration into host cell genomes that lead to insertion mutagenesis and genotoxicity for AAV vectors than for RV or LV vectors, which integrate into host cell genomes and lead to insertion mutagenesis [61]. Among the identified AAV serotypes (serotypes 1 to 13) that show a unique pattern of tissue tropism, AAV serotype 9 (AAV-9) can access the CNS by receptor-mediated transcytosis across the endothelium of BBB [95]. Therefore, the AAV-9 vector is applied more frequently in gene therapy for MPS [61].

Regenxbio designed the AAV9 vector to deliver the *α*-l-iduronidase gene for the treatment of MPS I (Project Code: RGX-111) and the iduronate-2-sulfatase gene for the treatment of MPS II (Project Code: RGX-121). In order to provide a permanent source of the secreted enzyme within the brain cells, intracisternal administration of RGX-111 in a phase I/II clinical trial with MPS I children is in progress to evaluate the safety and explorative effect (NCT03580083). A phase I/II trial of RGX-121 is designed for paediatric patients with MPS II who have or are at high risk of developing neurocognitive effects (NCT03566043) [62]. In July 2020, Regenxbio reported that RGX-111 and RGX-121 were well tolerated following one-time intracisternal administration, and data from a single patient with RGX-111 implied encouraging biomarker activity and continued progression in neurocognitive development. (REGENXBIO, PR Newswire, July 08, 2020).

In addition, site-specific *in vivo* genome editing, which is the genetic engineering tool used to modify DNA or RNA sequences, is under investigation. Genome editing is enabled by zinc-finger nucleases (ZFN) or clustered regularly interspaced short palindromic repeats-associated protein 9 (CRISPR/Cas9). These techniques have been thoroughly studied recently [96]. The engineered nuclease generates double-strand breaks (DSBs) at the correct position in the genome, which are repaired through nonhomologous end joining or homologous recombination. Sangamo Biosciences is developing an IV ZFN therapeutic for genome editing delivered by AAV vectors to insert a correct copy of the *α*-L-iduronidase gene or the iduronate 2-sulfatase gene into the genome of the patient's hepatocytes. The goal is to achieve life-long therapeutic production of the deficient enzyme for the treatment of MPS I (Project Code: SB-318) and MPS II (Project Code: SB-913). A phase I/II clinical trial designed to evaluate the safety and efficacy of SB-318 in three paediatric patients with MPS I (NCT02702115) and SB-913 in 9 paediatric patients with MPS II (NCT03041324) was initiated in 2017. However, a limited effect was observed in this study due to low transgene expression levels for both MPS I and MPS II (February 7, 2019/PRNewswire/Sangamo Therapeutics) [61, 62, 97]. It is hypothesized that delivering the defective genes directly into the brain via intracerebral or intra-CSF injection achieves better control over the neurological symptoms than systemic IV administration.

The approval of *voretigene neparvovec-rzyl* (Luxturna™, Spark Therapeutics), an AAV-2-vector that brings the correct copy of the RPE65 gene intended for the treatment of RPE65 mutation-associated retinal dystrophy, by the US Food and Drug Administration in December 2017, marked the beginning of a new era in medicine in which many inherited diseases will be corrected by gene therapy [98]. Gene therapy can control neurological and ophthalmic manifestations if engineered to target hard-to-reach systems such as the brain, bone, or eye by recombinant enzymes. Furthermore, in contrast to ERT which requires life-long weekly/biweekly injection with immunogenicity problems, gene therapy provides a one-time permanent treatment with permanent effect preservation. Although the risk of genotoxicity and lack of long-term safety are hurdles that need to be overcome, gene therapy is the most ideal strategy to benefit MPS patients in the future.

#### 2.3.3. Substrate Reduction Therapy (SRT)

SRT represents an alternative approach for the treatment of MPS. While ERT is aimed at eliminating GAG storage within the lysosomes, the goal of SRT is to partially inhibit or slow down the biosynthetic cycle and reduce substrate accumulation when the enzymes are malfunctioning. *Miglustat* (Zavesca®, Actelion Ltd.), the first SRT for lysosome disorders that slows down the production of glycosphingolipids, was approved in the European Union in 2002 to treat adults with mild-to-moderate type I Gaucher disease who were considered unsuitable for ERT [99]. As a synthetic analogue of D-glucose, *Miglustat* functions as a competitive inhibitor of glucosylceramide synthase, which is an essential enzyme in most glycosphingolipids synthetic chain. Later in 2009, *Miglustat* was approved for treating progressive neurological complications in people with Niemann-Pick disease type C. Several other SRT drugs are under development in preclinical and clinical studies for MPS patients.

Genistein (4,5,7-trihydroexyisoflavone), a natural isoflavone purified from soybean, showed GAG storage reduction via tyrosine kinase inhibition in MPS I, II, III, VI, and VII fibroblast cells and early clinical studies on MPS [100–102]. However, an open-label study with 19 MPS III paediatric patients treated with 5 mg/kg/day of genistein for 1 year failed to demonstrate improvement in the disability scale [103]. A double-blinded, randomized placebo-controlled clinical trial involving 30 patients with MPS III treated with 10 mg/kg/day of genistein showed small reduction in urinary GAG excretion and plasma HS, but no change in behaviour or hair morphology [104]. A phase III, double blinded, randomised, and placebo controlled clinical trial with a high dose (160 mg/kg/day) of oral administrated genistein for 1 year, followed by 1 year of open-label genistein administration, was initiated in 2014 (EudraCT 2013-001479-18). The result was reported in 2018 with slightly lower HS level in CSF. However, it failed to show any clinical benefit in MPS III patients [61].

Currently, Inventiva Pharma is developing an orally administrable small molecule called Odiparcil for the treatment of several types of MPS. Odiparcil, the *β*-1,4-galactosyltransferase (B4GalT7) decoy substrate, modifies GAG synthesis and facilitates the production of soluble GAG that can be excreted in the urine. It inhibits the accumulation of CS and DS in patients with MPS I, II, IVA, VI, and VII [105]. A 26-week, double-blinded, randomized, placebo-controlled phase IIa clinical study in patients with MPS VI older than 16 years of age was completed in September 2019 (NCT03370653). It displayed a good safety profile and improvements in cardiac and lung function and corneal clouding for patients receiving Odiparcil (News & Events, Inventiva, December 18, 2019). Following the positive results of a Phase IIa clinical study in adult patients, Inventiva is developing a phase I/II trial of Odiparcil in paediatric patients with MPS VI who are older than 5 years (News & Events, Inventiva, February 03, 2020).

Concurrently, BioMarin is developing a PIKFyve (FYVE finger-containing phosphoinositide kinase) inhibitor, which activates TFEB (transcription factor EB, a master transcriptional regulator of lysosomal biogenesis), TFE3 (transcription factor E3), and MITF (microphthalmia-associated transcription factor), thus enhancing lysosomal gene expression for MPS and other lysosome disorders (United States Patent Application Publication_US 2019/0249155 A1, Aug. 15, 2019).

SRT drugs are small molecules that are orally administrated that can easily access every organ/tissue to relieve GAG storage and disease manifestation. With the advantage of being distributed throughout the body, including the cartilage, eye, and brain, SRT may exhibit promising results on organ/tissues that are poorly managed by ERT or HSCT. Meanwhile, SRT could be combined with other therapies that target different tissue and stages in different disease progression status. In contrast to ERT or HSCT, SRT drugs are noninvasive. As a result, it is expected that SRT could be applied as a good strategy to control the unmanageable manifestations of neurological, musculoskeletal, and ophthalmological symptoms in the near future.

### 2.4. Supportive and Palliative Therapies

Considering the limitations of HSCT and ERT as disease-specific treatments, the management of symptoms with regular monitoring and supportive or palliative treatment is of utmost importance for MPS patients. Current supportive and palliative therapies include (1) surgical interventions, (2) physical therapy or occupational therapy, and (3) medications.  Table 3 summarizes the general management, including monitoring and treatment for uncontrolled symptoms of MPS I and MPS II [48, 59, 78].

Surgical interventions are required for various systemic symptoms and physical disabilities. Patients with MPS typically undergo surgical intervention at a very young age. Repeated surgical interventions are common in MPS patients. Data from the MPS I Registry, an international observational database, shows that about 75% of MPS I patients undergo the first surgery at less than 5 years of age [106]. A median of 3 to 4 operations was reported per patient, and the surgery percentages that preceded diagnosis were 36%, 46%, and 63% for patients with Hurler syndrome, Hurler-Scheie syndrome, and Scheie syndrome, respectively [54, 106]. Data from Hunter Outcome Survey, the multicenter observational database of MPS II, demonstrated that surgical interventions were performed in 83.7% of the MPS II patients, and patients underwent their first operation at a median age of 2.6 years. A median of 3 surgeries is performed in each MPS II patient [54]. The most common surgeries are tympanostomies/myringotomies, repair of inguinal hernias, adenoidectomy/tonsillectomy, and operations for carpal tunnel release. Valve replacement could be performed to manage cardiac valve manifestations that affect about half of the patients with MPS I and MPS II [107].

Rehabilitation therapy, including physical and occupational therapy, is required for patients with chronic physical disabilities and deformities to maintain physical function and activities of daily living. It provides a personalized treatment program according to each patient's symptoms and offers them a variety of treatment options. Approaches to enhance functional skills include gait training, static and dynamic balance, activities of daily living, and moving between positions. Physical therapy for children with MPS is to attain the developmental milestones and reach their full potential, and treatment is aimed at preventing future or present problems. Adults are more likely to have developed several complications. Hence, treatment in adults is carried out to maintain improvement, slow disease progression, and make the best use of preserved functions.

Previously approved medications can be used to alleviate symptoms. For example, standard agents are useful for cardiovascular manifestations. Hypertension could be treated using angiotensin-converting enzyme inhibitors, angiotensin receptor blockers, diuretics, and calcium channel blockers. Arrhythmias could be treated with antiarrhythmic drugs and anticoagulants [78].

#### 2.4.1. Management of Musculoskeletal Manifestations

While ERT or HSCT improves the life expectancy in children with MPS, in most cases, their efficacy comes into question because still struggled a long time with performing daily activities and significantly reduced walking duration [108]. Skeletal manifestations persist as the most common symptoms in MPS I and MPS II. Pain and stiffness within the muscles or joints and the physical appearance can negatively impact the quality of life in children [109]. Rehabilitation therapy is required to prevent complications and delays the progression of the disease [110, 111]. Patients should start at an early age to preserve functions for daily activity and slow symptom progression. As many MPS children present with delayed development and/or neurological regression, gain motor skills at a slow rate, and begin to lose motor skills as the disease progresses [112, 113], the Bayley Scales of Infant Development II (BSID-II) is widely used for motor function evaluation in children with MPS. However, the BSID-II does not allow the determination of separate scores for gross and fine motor function [114], and children with MPS tend to get higher scores in fine motor skills than in gross motor skills, which inflate the standard scores of BSID-II. Therefore, the Peabody Developmental Motor scale is recommended for the assessment of motor skills in children with MPS [113]. Physical therapists and occupational therapists can provide proper exercise or treatment after a comprehensive examination. In physical therapy, family involvement determines in children's developmental outcomes [115].

Typical unmanaged musculoskeletal symptoms in children with MPS I and MPS II include spinal abnormalities, hip dysplasia, genu valgum (knock knees), joint abnormalities, and abnormal gait. Kyphosis/scoliosis is a common spinal abnormality that results in pain, disability, cardiorespiratory complications, and even death [116]. Management of kyphosis/scoliosis involves physical therapy, usually applied for curves between 18° and 45° to straighten the spine as much as possible and avoid cardiopulmonary and neurological problems [116–118]. Although it is hard to prevent the progression of kyphoscoliosis, physical therapy can delay the postural collapse biomechanically that causes secondary functional impairment (such as restrictive pulmonary disease and reduced cardiopulmonary performance) and reduce pain [116, 119].

Hip dysplasia is characterized by an abnormality of the articular and periarticular structures and is defined by the instability of the hip, capsular laxity, or abnormal acetabulum. Orthopedic/physical therapy is important to correct hip position and articular angle and prevent further deterioration of the articular/periarticular structures. Diagnosis of hip dysplasia is established by reduced mobility movement, frontal pelvic asymmetry, higher contractures in the lumbar paravertebral muscles in the part of the hip with dysplasia, and difficulties in maintaining the prone position for more than 10 seconds. Physical therapy such as Swedish massage, posturing, and passive and active mobilization could be applied for regaining hip mobility/full range of motion, strengthening the hypotonic muscles, rebalancing the pelvic asymmetry, and increasing the passive and active stability of hip joints [120].

If joint stiffness or contracture is present, manual and active joint exercises could be applied to maintain or enhance range of motion (ROM). Subluxation is caused by the unalignment of the wing bone and clavicle and weakening or shortening of shoulder muscles, which can be managed by muscle strengthening or electrical stimulation. Joint ROM exercises may offer some benefits, so patients should start at an early age to preserve joint function and slow symptom progression. Repeated joint stretching during daily activities is beneficial to maintain joint motion. For example, stretching via long sitting or wedge standing while watching TV can helpful in addressing the knee ROM. Patients are advised to wear splints throughout the night while sleeping to maintain or regain the range of motion [121].

Genu valgum refers to abnormal knee alignment states in the frontal plane that cause problems in the overall alignment of the lower extremities and the knee joints and lead to osteoarthritis or knee deformities, increasing the risk of falls due to reduced postural stability [122, 123]. Genu valgum may involve the hip and the subtalar joint, as well as the patellofemoral joint, and treatment approaches should be applied accordingly. Adjustment of the joint and muscle alignment can be considered in some cases. Supportive devices such as orthotic footwear and walking aids can assist with daily living activities.

Abnormal gait is another problem encountered in children with MPS. Several studies have reported that strengthening exercise of lower extremities, balance exercise, and repetitive locomotor training may improve gait function [124–126]. When a child has difficulty walking/struggles with walking, the use of a standing frame or tilt-table can prevent osteoporosis or improve joint range of motion [127].

Hydrotherapy has been used for musculoskeletal and neuromuscular rehabilitation for more than 100 years, with improved motor performance in children with muscular dystrophy, cystic fibrosis, spina bifida, Rett syndrome, cerebral palsy, multiple sclerosis, and Parkinson's disease [128–131]. It is an effective yet enjoyable therapy for children with motor disabilities that can be administered to children with MPS I and MPS II, who are unable to perform certain activities on land [66, 132]. Hydrotherapy reduces excessive joint loading, enhances strengthening, and provides assistance to children with decreased postural control and muscle weakness [128, 131]. Buoyancy offers support to the joints and counteracts the gravitational force, which may facilitate postural control. Hydrostatic pressure provides different sensory feedback than land-based exercise, thus influencing balance competence and postural control [133]. Water resistance facilitates various forms of exercise, providing receptive resistance to muscle strengthening [131]. The warm temperature (33°C-35°C) reduces muscle spindle activity, promotes muscle relaxation, and reduces spasticity, which leads to an increased ROM in the joints and offers improved postural alignment [134]. It also provides the opportunity to experience, learn, and enjoy new movement skills, which result in increased functional skills and mobility, and builds self-confidence.

Surgical interventions for musculoskeletal manifestations include arthroscopy, hip or knee replacement, and correction of the lower limb axis. These orthopedic treatments can help address the psychosocial aspects of the disease, such as loss of mobility and independence [58, 70, 78]. Surgical intervention should be considered with caution, as the short neck, immobile jaw, and pathological changes in the airway found in patients with MPS make general anesthesia a challenging and high-risk procedure [78, 135].

#### 2.4.2. Management of Airway Abnormalities

Typical features of MPS include upper and lower airway obstruction and restrictive pulmonary disease, which can lead to chronic rhinosinusitis, recurrent upper and lower respiratory tract infections, and obstructive sleep apnea [136]. Upper airway obstruction may occur due to the deposition of GAGs in the soft tissues of the throat and trachea [135]. Patients with MPS should receive regular assessment for airway obstruction, and sleep studies are required in patients with sleep apnea [37, 135, 137]. Adenotonsillectomy, surgery of the nasal or shell, tracheostomy, and laser surgery of tracheal lesions are common surgical procedures for airway disorders in MPS patients [70]. The treatment of sleep apnea includes nocturnal supplemental oxygen. Although tonsillectomy and adenoidectomy may be performed when these are enlarged, temporary or partial improvement is observed due to the progressive disease character [54]. Respiratory support is useful for patients with manifestations of airway disease. Continuous positive airway pressure can be adopted to improve airway potency during sleep. It leads to significant improvements in sleep quality and a reduction in fatigue or headache complaints the following day [78, 135].

Respiratory physical therapy can be applied to patients with MPS to improve pulmonary ventilation and respiratory biomechanics. Upper airway obstruction, thoracic deformity, muscular shortening, protruding abdomen, and bronchoaspiration caused by dysphagia may lead to a reduction in abdominal thorax expansion and mobility, absence of productive cough, recurrent infections, and hypersecretion in MPS patients [108]. Pulmonary rehabilitation is a key component of managing obstructive airway symptoms, which involves exercise training, education, and self-management interventions [138]. Since patients exhibit reduction of thoracic volume and restriction of the diaphragmatic movement, increasing the flexibility of thoracic cage and strengthening the diaphragm muscle are needed [139]. Considering diaphragm is the main breathing muscle and patients suffer diaphragmatic weakness from spinal cord compression, it is important to perform diaphragmatic breathing and strengthen diaphragmatic muscles [140, 141]. Weakness of expiratory muscles and absence of/reduced productive cough lead to reduced airway clearance. Treatment options to assist airway clearance may include postural drainage, manual cough assistance, percussion, and vibration (chest clapping/shaking), a forced expiratory manoeuvre such as huffing, and an active cycle of breathing techniques [142]. Positive expiratory pressure (PEP) devices such as expiratory muscle strength training, TheraPEP, flutter, and acapella, high-frequency chest wall oscillation, and cough assist machine may be used for expiratory muscles strengthening and secretion clearance assistance. Blowing up a balloon or blowing out candles could be considered as a play therapy for MPS children.

#### 2.4.3. Management of Ocular Manifestations

Ocular involvement in MPS I generally consists of corneal clouding, retinopathy, glaucoma, and optic nerve abnormalities. Although MPS II is associated with similar ocular manifestations, corneal clouding resulting from the building up of GAGs (most likely HS) in stromal keratocytes is rarely encountered [12]. HSCT is reported to stabilize or improve corneal opacification, visual acuity, and optic nerve swelling [143], but is ineffective in preventing retinal degeneration [144]. ERT associates with stabilization of corneal clouding; however, the effects on visual acuity, optic nerve edema, or atrophy are inconclusive [12, 49]. Ocular evaluation and management should be comprehensive, and patients should receive monitoring every 6 to 12 months during preschool age and then every year until they reach the age of 18 [144]. Visual field tests are difficult to perform as most patients with MPS are very young with developmental delays [12].

The treatment of ocular complications in MPS does not differ substantially from approaches used for otherwise healthy individuals. Corneal transplants could be used to manage severe corneal clouding and restore corneal transparency [48]. However, reopacification occurs as early as 1 year after surgery due to GAG deposition in the graft without systemic treatment. Moreover, the graft visual acuity is often limited due to the secondary compromise of the retina and optic nerve [12]. Both penetrating keratoplasty (PK) and deep anterior lamellar keratoplasty (DALK) can be applied to patients. However, DALK is recommended over PK due to decreased risk of rejection [48]. Recently, a limbal stem cell transplant combined with keratoplasty is recommended to restore healthy limbal epithelial host cells and delay or prevent the recurrence of corneal opacification [12, 49, 145].

Management of glaucoma is difficult due to the limited effectiveness of antiglaucoma therapy and the progressive disease characteristics [12]. GAG accumulation in the trabecular meshwork and aqueous outflow pathways can lead to glaucomatous changes in the optic nerve, seen in other forms of open-angle glaucoma pathophysiology. GAGs depositions in the peripheral cornea, other anterior chamber structures, and cystic changes in the ciliary body may lead to closed-angle glaucoma. Notably, measured elevated intraocular pressure (IOP) may be inaccurate for MPS patients [48]. Patients may have falsely elevated IOP due to the increased corneal rigidity. For a thorough evaluation of glaucoma, advanced technologies such as ultrasound biomicroscopy and anterior segment optical coherence tomography that can visualize the anatomy behind the potentially hazed cornea could be used for diagnosis and monitoring. Patients diagnosed with glaucoma may benefit from the use of IOP-lowering eye drops.

Management of retinopathy is challenging, as no positive effects have been observed with ERT or HSCT. Patients with MPS may exhibit retinopathy with pigmentary retinal degeneration and associated electroretinogram changes due to GAG deposition in the retinal pigment epithelial cells, resulting in photoreceptor loss. For corneal clouding or photophobia in MPS patients, the diagnosis of retinopathy requires a comprehensive examination with fundus photography or echography for the assessment of the optic nerve and retinal pathologies [48]. There is no treatment available for optic nerve involvement associated with retinal degeneration. However, as previously mentioned, the approval of Luxturna™ as gene therapy for RPE65 mutation-associated retinal dystrophy brings hope to MPS patients. Considering the sustained expression and action of *β*-glucuronidase in MPS VII canine retinal pigment epithelium transduced with AAV virus vector containing *β*-glucuronidase cDNA *in vitro* [146], successful management of retinal pathology in MPS will be realized in the future.

#### 2.4.4. Management of Neurological Manifestations

Nerve conduction studies should be performed every 1 to 2 years, from 4 to 5 years of age, to monitor peripheral nerve function and the development of carpal tunnel syndrome [59, 66, 144]. Carpal tunnel syndrome is a rare finding in healthy children but frequently reported in children with MPS I and MPS II [147, 148]. Patients have distorted bony architecture in the distal wrist, including distal radio-ulnar dislocation, small irregular carpal bones, and short tubular metacarpals with decompression of the median nerve. All these physiological abnormalities lead to loss of sensation or abnormal nerve conduction [149]. Decompression surgery should be performed for patients who suffer from carpal tunnel syndrome with loss of hand sensation or function [59]. Spinal cord compression, which results in cervical myelopathy, occurs due to cervical stenosis, thoracolumbar kyphosis/scoliosis, and lumbar canal stenosis [150]. It should be addressed by depression surgery before irreversible cord damage occurs [137, 151, 152]. It is important to detect spinal cord compression as early as possible before an irreversible loss of motor function or sensation in all limbs occurs [59]. After decompression surgery, several methods have been introduced, namely, *in situ* fusion using a halo-vest, cables or wires, transarticular screws, or laminar screws. A halo-vest could stabilize the head and neck during and after the surgery in children with MPS, whereas Mayfield fixators are used for adults [150]. A collar can be used by MPS children with mild symptoms immediately after surgery. Children can switch to a neck restraint or collar after 3 to 6 months of wearing halo-neck braces.

Previously approved standard medications can be used to alleviate symptoms of pain, seizures, sleep disorders, and psychiatric problems. Anticonvulsant therapy can reduce the frequency of seizures and may improve sleep, cognitive, and behavioral symptoms [55, 137, 153]. Although antipsychotic agents and attention stimulants may improve behavioral disorders associated with MPS II, the prescription should be used with caution due to the limited number of reports on the efficacy of the reagent [59]. Given the cognitive impairment and developmental delay in severe types of MPS I and MPS II, it is important to provide MPS children with a stimulating learning environment to achieve learning and normal developmental milestones as early as possible before deterioration occurs in the later stage. Special schooling or speech therapy is recommended for patients with cognitive impairments or behavioral problems [59].

## 3. Conclusions

MPS I and MPS II show a wide spectrum of clinical manifestations and disease severity. A comparison of the natural history illustrates that the onset of both somatic and central nervous system manifestations occur earlier in severe type of MPS I than in MPS II. Although HSCT and ERT have advantages in alleviating soft tissue-related concerns and offer improvements in walking distance, forced vital capacity, respiratory symptoms, cardiovascular symptoms, and hepatomegaly, these therapeutic modalities are ineffective for brain-related dysfunction, bone deformity, and optic nerve disorders.

Many pharmaceutical companies are developing BBB-penetrable ERT to control CNS manifestations currently uncontrolled with ERT and overcome the limitations of ERT and HSCT. The development strategy is to conjugate the BBB-targeting antibody or sequence to approved ERT drugs and enable drug delivery into the brain. As this approach is in the late stages of clinical trials, it is expected that BBB-penetrable ERT will be commercialized within next few years. Secondly, orally available SRT drugs would take some more time for commercialization. SRT is expected to address the shortcomings of ERT, such as its limited effect due to distribution problem, and provide solutions for the unmanaged manifestations of neurological, musculoskeletal, and ophthalmological symptoms. Lastly, gene therapy exhibits the advantage of producing life-long effects with one or two administrations. It is an ideal strategy to “cure” the disease rather than support by “care.” Although gene therapy has shown promising results in preclinical and clinical studies, it requires some more time to show benefits in MPS patients. Gene therapies are thoroughly studied in early clinical stages of development and need to overcome safety issue such as genotoxicity and long-term safety. Some clinical studies reveal that the delivery of genes directly into the brain provides better control of neurological symptoms than intravenous systemic administration.

**Figure 1:**
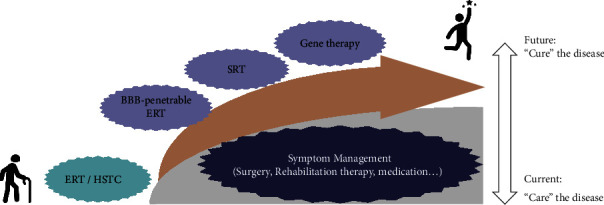
Therapeutic options and management strategy of MPS I and II.

In these circumstances, it is important to manage the disease with regular follow-ups and supportive/palliative therapy to relieve the symptoms (Figure 1). Surgical interventions, physical therapy, hydrotherapy, and symptomatic medications could be considered for patients with MPS I and MPS II. Musculoskeletal manifestations can be managed by orthopedic surgery, including arthroscopy, hip or knee replacement, and correction of the lower limb axis, even before disease diagnosis. Physical and occupational therapy provide personalized exercise and treatment for patients (according to their status) to prevent or improve musculoskeletal abnormalities. Hydrotherapy, which is widely used for musculoskeletal and neuromuscular rehabilitation and demonstrates improved motor performance in many diseases, could be applied to MPS patients as play therapy. To manage airway abnormalities, respiratory physical therapy that is aimed at improving the pulmonary ventilation and respiratory biomechanics may benefit patients. Continuous positive airway pressure could be applied for patients with sleep problems. Treatment of ocular complications in MPS patients does not differ substantially from approaches used for otherwise healthy individuals. With the advent of gene therapy, successful management of retinal pathology in MPS I and MPS II will be realized in the near future. For carpal tunnel syndrome and spinal cord compression, decompression surgery should be performed as early as possible before an irreversible loss of motor function or sensation occurs. Standard medications can be used to alleviate neurological symptoms, such as pain, seizures, sleep disorders, and psychiatric problems. Providing a stimulating environment for children living with MPS and allowing them to achieve learning and normal developmental milestones are crucial.

In summary, health care providers should consider the unique disease characteristics and manifestations of each patient carefully before prescribing any treatment modalities. In addition, they can suggest appropriate supportive and palliative therapies that benefit their patients most.

## Figures and Tables

**Table 1:** Prevalence and onset age of typical manifestations in severe type MPS I and MPS II.

Clinical symptoms	MPS I		MPS II	
	Prevalence	Onset years of age ^∗^	Prevalence	Onset years of age ^∗^
Coarse face	86.4%	0.9 years	95%	2.4 years
Hepatosplenomegaly	70.0%	1.1 years	89%	2.8 years
Hernias	58.9%	0.8 years	78%	1.3 years
Airway abnormalities^†^	41%	1.2 years	70%	3.4 years
Cardiac valve disease	48.9%	1.3 years	57%	6.1 years
Skeletal disorder				
Joint contractures	37.9%	1.6 years	84%	3.6 years
Kyphosis/scoliosis	70.0%	1.0 years	33.8%	6.4 years
Dysostosis multiplex	44%	1.0 years	8.6%	N/A
Spinal stenosis	N/A	N/A	46%	14.3
Ocular manifestation				
Corneal clouding	70.9%	1.1 years	Not common	N/A
Retinopathy	80%	10 years		<21 years
Glaucoma	10%	1 years		7.5 years
Neurological disorder				
Seizure	29%	N/A	18%	9.3 years
Carpal tunnel syndrome	7.8%	2.3 years	25%	7.9 years
Brain abnormalities^††^	NR	Most are evident at <2 years	NR	6.0 years^∗∗^
Cognition impairment	46.4%	1.2 years Plateaus at 3 years, then decline	100%	3.2 years^∗∗^ Plateaus at 4 years, then decline
Behavioral problem	65%	N/A	73%	Hyperactive and aggressive ±4 years Sleeping problems 4.3 years

^∗^Given ages represent approximants of median ages in rapidly progressing MPS types, unless indicated otherwise. ^∗∗^Given as mean ages; ^†^airway disease due to enlarged tongue, tonsils, and restrictive lung disease caused by inefficient mechanical properties of the chest. ^††^Brain abnormalities include symptoms of hydrocephalus, ventriculomegaly, enlargement of perivascular spaces, and atrophy.

**Table 2:** New drug candidates target unmanaged manifestations in MPS I and MPS II.

Strategy	Type	Drug name	Stage	Administration	Mode of action	Sponsor	Reference
BBB-penetrable ERT	MPS I	Iduronidase-IT	Ph I	IT	CNS administration	Shire	NCT00638547
		AGT-181	Ph I/II	IV	Insulin receptor-mAb conjugated enzyme	ArmaGen	NCT03053089
		JR-171	Ph I/II	IV	Transferrin receptor-mAb conjugated enzyme	JCR Pharmaceuticals	NCT04227600
		GNeo-IDUA	Pre-IND	IV	GNeo-conjugated enzyme	TEGA therapeutics	[91]..
	MPS II	Idursulfase-IT	Ph II/III	IT	CNS administration	Shire	NCT02055118
		Idursulfase-beta-ICV	Ph I/II	ICV	CNS administration	GC Pharma	[87]..
		JR-141	Ph II/III	IV	Transferrin receptor-mAb conjugated enzyme	JCR Pharmaceuticals	NCT03568175
		AGT-182	Ph II	IV	Insulin receptor-mAb conjugated enzyme	ArmaGen	NCT02262338
		DN-310	Ph I/II	IV	Transferrin receptor-mAb conjugated enzyme	Denali therapeutics	NCT04251026
Gene therapy	MPS I	RGX-111 (AAV9-IDUA)	Ph I/II	ICS	In vivo gene delivering with AAV	Regenxbio	NCT03580083
		SB-318 (AAV-ZFN)	Ph I/II	IV	ZFN mediated genome editing	Sangamo therapeutics	NCT02702115
	MPS II	SB-913 (AAV-ZFN)	Ph I/II	IV	ZFN mediated genome editing	Sangamo therapeutics	NCT03041324
		RGX-121 (AAV9-IDS)	Ph I/II	ICS	In vivo gene delivering with AAV	Regenxbio	NCT03566043
Substrate reduction therapy	Pan-MPS	Genistein	Ph III	Oral	Reduces proteoglycan biosynthesis	Manchester University	2013-001479-18†
		Odiparcil	Ph IIa	Oral	B4GalT7 decoy substrate	Inventiva Pharma	NCT03370653
		PIKFyve inhibitor	Discovery	N/A	Enhance lysosomal gene expression	Biomarin	US 2019/0249155††

IT: intrathecal; IV: intravenous; ICV: intracerebroventricular; ICS: intracisternal; IC: intracerebral; AAV: adeno-associated virus; ZFN: zinc finger nuclease; IDS: iduronate-2-sulfatase; IDUA: alpha-l-iduronidase; B4GalT7: *β*-1,4-galactosyltransferase; PIKFyve: FYVE finger-containing phosphoinositide kinase; GNeo: guanidinylated form of neomycin; mAb: monoclonal antibody. ^†^EU Clinical Trials Register number, ^††^United States Patent Application Publication_ Pub. No.: US 2019/0249155 A1, Pub. Date: Aug. 15, 2019.

**Table 3:** Management of unmanaged symptoms in MPS.

Clinical symptoms	Monitoring^a,b,c,g^	Treatment ^a,b,c,d,e,f,g^
Cardiac valve disease	Echocardiogram; cardiac MRI	Value replacement
Recurrent ear infections, hearing loss	Otological and audiological examinations	Grommet; hearing aids
Airway obstructions	Upper airway examination; sleep studies	Respiratory physical therapy; positive airway pressure ventilator
Hernias	Clinical examination	Surgery
Joint/Skeletal muscular manifestations		
Joints contraction	6-minute walk test; joint range of motion	Physical therapy; splints
Kyphosis/scoliosis
Hip dysplasia	Clinical examination	Surgery; physical/occupational therapy
Genu valgum		
Abnormal gait		
Ocular manifestations		
Corneal clouding	Clinical examination	Contact lenses; corneal transplant
Glaucoma		Pressure-lowering eye drops
Retinopathy		N/A
Neurological manifestations		
Cognitive impairment	Neurobehavioral assessment; cognitive testing	Stimulating environments; special schooling; speech therapy
Behavioral problems	Aim to rule out comorbid conditions	Antipsychotics stimulants; mood stabilizer; behavioral therapy
Seizures	Brain MRI; EEG	Anticonvulsant therapy
Hydrocephalus	Brain MRI	Ventriculoperitoneal shunting
Carpal tunnel syndrome	Nerve conduction studies; wrist ultrasound	Decompression surgery
Spinal cord compression	Spine MRI; somatosensory evoked potential	Decompression surgery, fixation (e.g., halo)

^a^Joseph Muenzer et al. (2008), “Mucopolysaccharidosis I: Management and Treatment Guidelines.” ^b^Ana Maria Martins et al. (2009), “Guidelines for the Management of Mucopolysaccharidosis Type I.” ^c^Maurizio Scarpa et al. (2011), “MPS II European recommendations for the diagnosis and multidisciplinary management of rare disease.” ^d^Hernan Amartino(2015), “Hunter Syndrome (Mucopolysaccharidosis II) – The Signs and Symptoms a Neurologist Needs to Know.” ^e^Sun H. Peck et al. (2016), “Pathogenesis and Treatment of Spine Disease in the Mucopolysaccharidosis.” ^f^Maurizio Scarpaa et al. (2017), “Epilepsy in mucopolysaccharidosis disorders.” ^g^Shizuka Tomatsu et al. (2019), “Ophthalmological Findings in Mucopolysaccharidoses”.

## Data Availability

No data were used to support this study.

## Supplementary Materials

Supplementary Materials Figure S1: degradation process of GAG chains and enzyme malfunction in each step. Table S1: deficient enzymes, accumulated substrate, and incidence of each MPS type.
