# Supplementary material for: Mucopolysaccharidoses I and II: Brief Review of Therapeutic Options and Supportive/Palliative Therapies
Source: Biomed Res Int. 2020 Dec 4;2020:2408402. doi: 10.1155/2020/2408402 (PMC7732385; doi:10.1155/2020/2408402)
Supplement: Supplementary Materials — Figure S1: degradation process of GAG chains and enzyme malfunction in each step. Table S1: deficient enzymes, accumulated substrate, and incidence of each MPS type. [file 2408402.f1.docx]

**Figure S1. Degradation process of GAG chains and enzyme malfunction in each step**


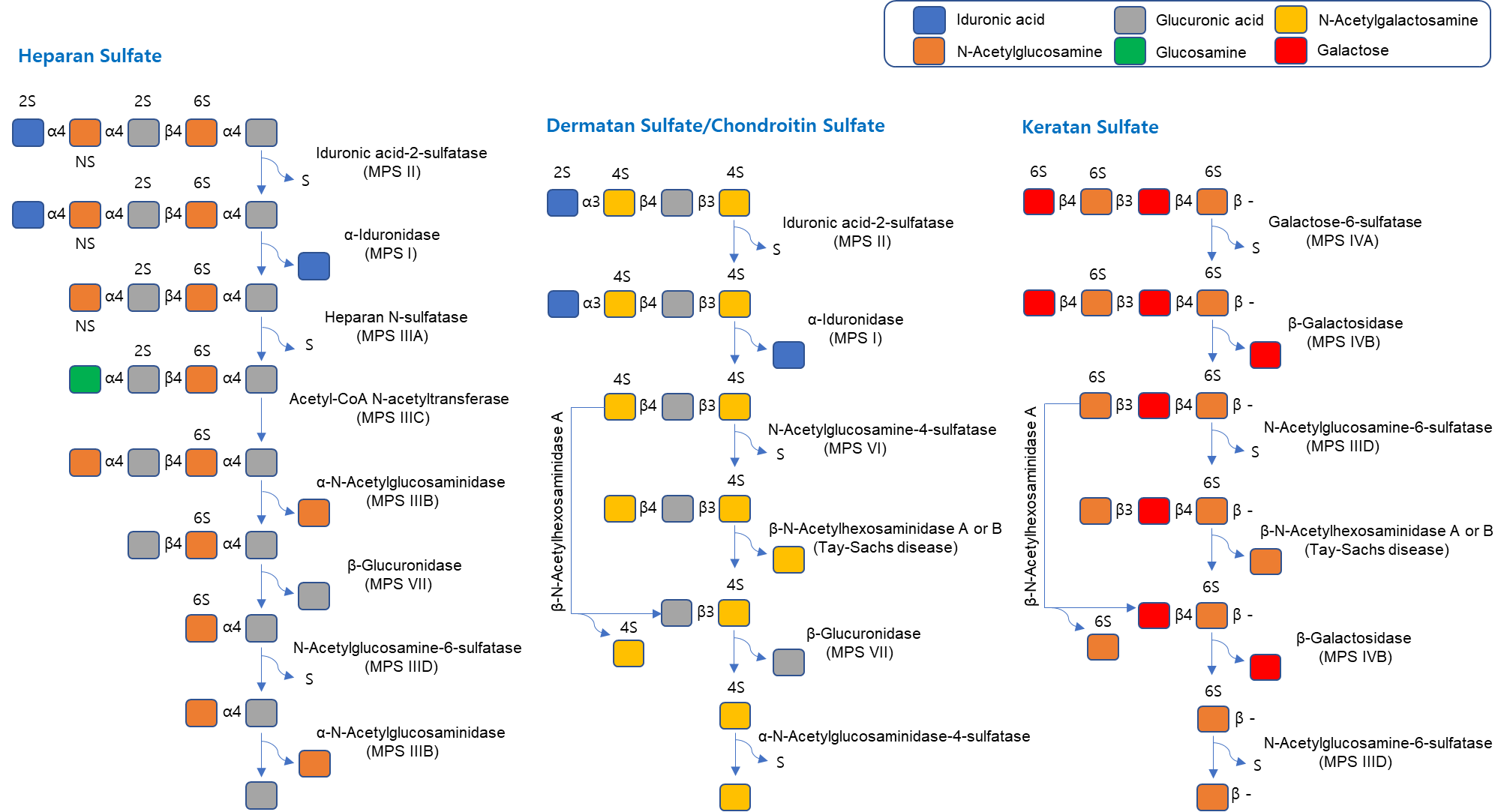


| MPS Type | | | Deficient Enzyme | | Gene locus | Genetic inheritance | GAG  Storage | Neurological symptoms† | Somtaic  Symptom† | Incidence†† |
| --- | --- | --- | --- | --- | --- | --- | --- | --- | --- | --- |
| MPS I  (Hurler/Hurler-Scheie/Scheie syndrome) | | | | α-L-iduronidase | 4p16.3 | AR | HS, DS | + /– | + | 0.69-1.66 |
| MPS II  (Hunter syndrome) | | | | Iduronate-2-sulfatase | Xq28 | XR | HS, DS | + /– | + | 0.30-0.71 |
| MPS III  (Sanfillippo syndrome) | A | Heparan-N-sulfamidase | | | 17q25.3 | AR | HS | ++ | – | 0.27~1.89 |
|  | B | α-N-acetylglucosaminidase | | | 17q21 |  |  |  |  |  |
|  | C | AcetylCoA-α-glucosaminide N-acetyltransferase | | | 8p11.1 |  |  |  |  |  |
|  | D | N-acetylglucosamine-6-sulfatase | | | 12q14 |  |  |  |  |  |
| MPS IV  (Morquio syndrome) | A | N-acetylgalactose-6-sulfate | | | 16q24.3 | AR | KS, CS | – | + | 0.22-1.30 |
|  | B | β-galactosidase | | | 3p21.33 |  |  |  |  |  |
| MPS VI  (Maroteaux-Lamy syndrome) | | | N-Acetylgalactosamine-4-lsulfatase | | 5q11-q13 | AR | DS, CS | – | + | 0.36-1.30 |
| MPS VII  (Sly syndrome) | | | β-D-glucuronidase | | 7q21.11 | AR | HS, DS, CS | + /– | + | 0.05-0.29 |
| MPS IX  (Natowicz syndrome) | | | Hyaluronidase | | 3p21.3-21.2 | AR | Hyaluronan | – | + | N/A |

**Table S1. Deficient enzymes, accumulated substrate, and incidence of each MPS type**

†Symptom presenceation: **+** present; **–** absent

††Disease incidence per 100,000 live births, AR: Autosomal recessive disorder, XR: X-linked recessive disorder
